# Diagnosis of Malignancy in Thyroid Tumors by Multi-Layer Perceptron Neural Networks With Different Batch Learning Algorithms

**DOI:** 10.5539/gjhs.v7n6p46

**Published:** 2015-03-30

**Authors:** Saeedeh Pourahmad, Mohsen Azad, Shahram Paydar

**Affiliations:** 1 Colorectal Research Center, Shiraz University of Medical Sciences, Shiraz, Iran; 2 Department of Biostatistics, Shiraz University of Medical Sciences, Shiraz, Iran; 3 Trauma Research Center, Department of surgery, Shiraz University of Medical Sciences, Shiraz, Iran

**Keywords:** thirteen batch learning algorithms, daignosis of malignancy, thyroid tumor, Accuracy in prediction, ROC curve

## Abstract

To diagnose the malignancy in thyroid tumor, neural network approach is applied and the performances of thirteen batch learning algorithms are investigated on accuracy of the prediction. Therefore, a back propagation feed forward neural networks (BP FNNs) is designed and three different numbers of neuron in hidden layer are compared (5, 10 and 20 neurons). The pathology result after the surgery and clinical findings before surgery of the patients are used as the target outputs and the inputs, respectively. The best algorithm(s) is/are chosen based on mean or maximum accuracy values in the prediction and also area under Receiver Operating Characteristic Curve (ROC curve). The results show superiority of the network with 5 neurons in the hidden layer. In addition, the better performances are occurred for Polak-Ribiere conjugate gradient, BFGS quasi-newton and one step secant algorithms according to their accuracy percentage in prediction (83%) and for Scaled Conjugate Gradient and BFGS quasi-Newton based on their area under the ROC curve (0.905).

## 1. Introduction

In recent years, digital revolution makes a huge volume of information collected and stored. Especially in health information, large databases of patient’s findings are already available. Data mining methods are powerful tools to assist physicians in decision making. These methods model the relations among clinical findings and hence help the physicians in diagnosing similar cases. However, the final decision will be still up to the doctor (Raghavendra & Srivatsa, 2011). For instance, logistic regression method is a probabilistic classification technique. It models the relationship between a binary outcome (healthy/unhealthy or death/survival) and a set of related attributes (risk factors). The derived model then helps the physician in prediction, diagnosis and treatment of the diseases during a reasonable time (Raghavendra & Srivatsa, 2011). This statistical method also depends heavily on its theoretical underlying assumptions (Pourahmad, Ayatollahi, & Taheri, 2011). Therefore, its flexibility in adapting to real data circumstances is reduced. But it is a powerful method with simple interpretations if its assumptions are met. However, the nature of clinical findings and large number of attributes under consideration require more flexible methods with no theoretical assumptions. Neural networks method is such the methods. It is able to model the complex relations among a large data set without any theoretical assumptions. Therefore, it can be a useful tool for modeling the relation among clinical findings (Amato et al., 2013).

Simple neural network was firstly introduced in 1943 (McCulloch & Pitts, 1943). Since then many developments have been occurred in its theory and applications. In theory for instance, different learning methods and diverse training algorithms have been proposed. The applications of neural networks in diverse fields such as clinical researches are also attractive. Among the recent studies, cancer diagnosis (Bourdes & Bonnevay, 2010), disease diagnosis (Alizadehsani et al., 2013), death prediction (Shi et al., 2012) and image classification (Kuruvilla & Gunavathi, 2014) can be mentioned. However, there are few researches for investigating the performance of different learning algorithms on accuracy of the results.

Thyroid nodule is a common problem in human population. Therefore, decision making for its management is controversial. Its management varies from observation to total thyroidectomy. To determine the type of management, Fine Needle Aspiration (FNA) of the nodule is one of the most useful tools. Indeed, it determines the type of surgery. If the test detects a benign tumor, then right, left or subtotal lobectomy is applied. Otherwise, total lobectomy is performed. However, clinical texts report some limitations in accurate report and some significant mistakes while decision is made based on FNA result (Zhang & Berardi, 1998). Among the affective factors on malignancy in thyroid tumors, there are factors such as age, gender, size of thyroid gland, tumor size, type of operation, type of malignant tumor, malignant tumor size, duration of the disease and family history. To detect the importance of these factors in diagnosis of malignancy, it is necessary to search fully in patients’ attributes to find and model the meaningful relations among their clinical findings. Hence the diagnosis process may be developed.

Accordingly, in present study, three neural networks with one hidden layer and 5, 10 and 20 neurons are considered. The performances of thirteen different batch learning algorithms are then compared in diagnosis of malignant thyroid tumors. The superior algorithms are chosen based on accuracy percentage in prediction and area under the ROC curve.

## 2. Methods and Materials

### 2.1 Materials

This study includs all patients who were initially diagnosed for thyroid tumors surgery in both sexes and all age groups. FNA test was performed on them and they were operated in Shahid Rajaee and Nemazee hospitals (two hospitals in Shiraz, southern Iran) during 2009 to 2012. The number of eligible people for the study was 345 persons. Based on clinical expert opinion, all factors related to type of thyroid tumors (malignant/benign) before the surgery were collected from the patients’ hospital records. Accordingly, 12 important factors such as gender, age, type and growth of the thyroid gland, FNA test result, duration of disease, family history of disease and cancer, size of the right and left thyroid gland and size of nodules in the left and right thyroid glands were considered in the modeling process.

### 2.2 Methods

In a classic definition, artificial neural network is a large set of parallel processors with a natural talent for storage of experimental data. It is like the brain for at least two stages: synaptic weights to store knowledge and a process called learning (Reggia, 1993).

In present study, a supervised network known as feed-forward neural network (FNN) will be applied with back-propagation (BP) training algorithm. One hidden layer with three different numbers of neurons including 5, 10 and 20 neurons is considered and thirteen batch learning algorithms for training the network are compared.

To recall the different activation functions or learning algorithms in neural networks modeling process, their abbreviations in MATLAB software will be presented in parenthesis in the following sections.

### 2.3 BP Algorithm

FNNs are applied to approximate the non-linear complex functions and hence are appropriate to model the ambiguous relations among clinical findings. The BP algorithm is a frequently used learning algorithm for training FNNs with high modeling power. It adjusts the network parameters iteratively to minimize the sum of squared approximation errors using a gradient descent technique (Sibi, Jones, & Siddarth, 2013).

The learning steps in this algorithm are as follows (Raghavendra & Srivatsa, 2011):

1) Inputs are entered into the system and go ahead trough the network layers with forward method until the output layer is reached. Then the output is predicted by considering the initial values for the parameters (weights and biases).
2) The network errors are defined as the difference between the predicted output and the target output.
3) Then it goes back and tries to decrease the errors by adjustment of the weights. Therefore, the mean square deviation between the predicted and target outputs is minimized in this method.
4) These steps are repeated reciprocally until the errors between the predicted and the actual outputs are minimized.

### 2.4 Activation Functions

Activation function is a linear or non-linear function which is applied on the outputs of the previous layer to build the inputs of the next layer. It would be possible to use different activation functions for each layer and even for each neuron in a layer.

Generally, in BP FNNs just three activation functions namely Linear (purelin), Log-Sigmoid (logsig) and Hyperbolic Tangent Sigmoid (tansig) can be used since these functions are differentiable (Hagen, Demuth, & Beale, 1996). As mentioned, the outputs in our clinical dataset were dichotomous. The bipolar data representation was then used for the target outputs (malignant: 1 and benign: -1). Generally, the binary data representation leads to elimination (to be zero) the network’s coefficients and consequently affects the learning process. Indeed, zero units are not learned (Fausett, 1994). As a result, the appropriate activation function was tansig for two conjunctions (input-hidden and hidden-output layers) in our study.

### 2.5 Batch Learning Algorithms

When the number of layers, the number of neurons and the activation functions in each layer are determined, the method of parameters’ adjusting (learning algorithm) should be chosen. There are two different learning algorithms namely ’sequential or online’ and ’batch’ learning methods (MATLAB, 2010a). In sequential learning method, parameters are updated after applying each pattern (instance) to the network. But in batch training algorithm, the updating process is performed after applying all patterns.

In present study, batch learning algorithm was used and thirteen different methods in this algorithm which are available in MATLAB software were compared. These methods are summarized as follows:

I) Basic Gradient Descent (traingd): Weights and bias in this method are updated in the opposite direction by the slope of the activation function. It responds slowly and can be used in sequential (incremental) mode training.
II) Gradient Descent with Momentum (traingdm): The direction of slope’s and errors’ changes is both considered in this method. It converges faster than the basic gradient decent algorithm and can be used in both learning mode.
III) Gradient Descent with Adaptive (traingda): The learning rate is changed based on the efficiency of the algorithm.
IV) Resilient Back propagation (trainrp): It eliminates the detrimental effects on the size of the partial derivatives and uses just the sign of derivatives to determine the direction of the weights’ update. It is a simple batch mode training algorithm with fast convergence and minimal storage requirements.
V) Fletcher-Powell conjugate gradient (traincgf): It updates the weight and bias values according to the conjugate gradient BP with Fletcher-Reeves updates. It has smallest storage requirements of the conjugate gradient algorithms.
VI) Polak-Ribiere conjugate gradient (traincgp): It updates the weight and bias values according to the conjugate gradient BP with Polak-Ribiere updates. It needs slightly larger storage requirements than traincgf and converges faster on some problems.
VII) Powell -Beale conjugate gradient (traincgb): It updates the weight and bias values according to the conjugate gradient BP with Powell-Beale restarts. It requires slightly larger storage requirements than traincgp and has a generally faster convergence.
VIII) Scaled Conjugate Gradient (trainscg): It is a combination of conjugate gradient and Levenberg – Marquatdt algorithms which avoids the time consuming linear search. It is the only conjugate gradient algorithm that requires no line search and is a very good general purpose training algorithm. Conjugate gradient algorithm does not require the computation of second derivatives and uses linear search to converge to minimum mean square deviation after a finite number of iterations.
IX) Levenberg – Marquatdt (trainlm): It is a popular curve-fitting algorithm used in many software applications for solving generic curve-fitting problems and finds only a local minimum. It is the fastest training algorithm for networks of moderate size and has memory reduction feature for use when the training set is large.
X) BFGS quasi-Newton (trainbfg): It updates weight and bias values according to the BFGS quasi-Newton method and requires storage of approximate hessian matrix. It has more computations for iterations than conjugate gradient algorithms, but usually converges in less iteration.
XI) One step secant (trainoss): It updates the weight and bias values according to the one step secant method and compromises between conjugate gradient methods and quasi-Newton methods.
XII) Gradient descent with momentum and adaptive learning rate (traingdx): It updates the weight and bias values according to gradient descent momentum and an adaptive learning rate. It has faster training than traingd, but it can only be used in batch mode training.
XIII) Bayesian regularization (trainbr): It updates the weight and bias values according to Levenberg-Marquardt optimization. It modifies the Levenberg-Marquardt training algorithm to produce networks that generalize well and reduces the difficulty of determining the optimum network architecture.

In all mentioned algorithms, training stops when any of these conditions occurs: The maximum number of epochs (repetitions) is reached or the maximum amount of time has been exceeded.

### 2.6 Performance Evaluation of Modeling Methods

#### 2.6.1 Receiver Operating Characteristic Curve (ROC curve)

ROC curve is used to evaluate discriminating power of the different methods especially for comparing diagnostic tests (Shang, Lin, & Goetz, 2000). Whenever the method or the system fails to recognize (diagnose) a disease correctly, the curve is a straight line between the points (0,0) and (1,1) in a two-dimensional space. While the performance of the method is accurate, its ROC curve is a vertical line between the points (0,0) to (0,1) and then a horizontal line to the point (1,1). Usually, the curves of different methods lie between these two positions unless the performance of the method or system is weaker than a random prediction. Area under the ROC curve also represents relative performance of the method. The amount of 0.5 indicates no apparent accuracy and the amount of 1 shows perfect accuracy (Shang, Lin, & Goetz, 2000). A nonparametric statistical method was used to test the significance difference of this area from the value 0.5.

#### 2.6.2 The Accuracy Percentage in Prediction

This value is calculated by cross-validation method. In training process, dataset is divided into k separate parts (k-fold). Then *k-1* parts are applied for model construction (training the system) and the remaining part is used to test the model. In testing process, the best model among k different models is chosen. The accuracy rate in each tour is defined as the number of correct predicted patterns divided by total number of patterns multiplied by 100.

## 3. Results

In this study, a BP FNN with one hidden layer was applied with ’tansig’ activation function in both layers. The activation function was considered the same for all neurons in each layer. Furthermore, three different numbers of neuron in hidden layer including 5, 10 and 20 neurons were compared. Moreover, thirteen different training methods in batch learning algorithm (explained in section 2.1.3) were applied to train the network. Table 1 summarizes the general characteristics of the network.

**Table 1:** General characteristics of FNNs in present study

Hidden layers number	1
Neurons in hidden	5, 10, 20
layer Neurons in output layer (decision classes)	1
Inputs number (quantitative and indicator variables)	10
Learning algorithm	13 mentioned batch learning method
Learning Rate	0.1
(difference between two adjacent error components)	0.05
Number of tours	20
Max Iterations	5000
Validation method	10-fold
The objective function	MSE (Mean squared error)
Size of training set	276(80%)
Size of validation set	69(20%)

As mentioned earlier, clinical findings of 345 patients with thyroid tumor referred for surgery were used for training the networks. Table 2 describes the patients’ attributes used as the system inputs and output. True status of the tumor (malignant or benign) determined by pathological result after surgery was used as target output. The result of FNA test as a preoperative diagnostic criterion along with other clinical findings of the patients before surgery was considered as the inputs. The result of FNA test compared with actual tumor type after surgery in present study shows 63 percent accuracy in diagnosis which is in agreement with other clinical texts (Raghavendra & Srivatsa, 2011).

**Table 2:** Description of patients’ attributes

Attributes	Statistical description
**Inputs**	No. (%)
**Qualitative**	
Gender	
Man	66(19.1)
Woman	279(80.9)
Having multiple nodules
Yes	182(52.8)
No	163(47.2)
Having fast growth of thyroid gland
Yes	251(72.8)
No	94(27.2)
Family history of thyroid disease
Yes	60(17.4)
No	285(82.6)
Family history of cancer in general
Yes	61(17.7)
No	284(82.3)
FNA test result
Benign	173(50.1)
Malignant	172(49.9)
**Quantitative**	Mean (SD)
Age (year)	40.9 (13.4)
Maximum size of the right thyroid gland (cm)	5.2(2.7)
Maximum size of the left thyroid gland (cm)	4.7(2.7)
Maximum size of nodules in the right thyroid gland (cm)	1.1(1.5)
Maximum size of nodules in the left thyroid gland (cm)	1(1.6)
Duration of disease (year)	4.2(3.3)
**Output**	No. (%)
Tumor type after surgery
Benign	189(54.8)
Malignant	156(45.2)

Dataset was randomly divided into two parts: 80 percent (276 cases) as the training set for learning and 20 percent (69 cases) as the unseen data for validation. In each thirteen training algorithms, training dataset was randomly divided into two separate parts. Minimum (Min), maximum (Max), mean and standard deviation (SD) values of accuracy percentages in diagnosis were then calculated for each algorithm (Table 3).

**Table 3:** Prediction accuracy of thirteen learning algorithms on validation data

Learning algorithm	Number of tours	Percentage of accuracy in prediction								

		5 neurons			10 neurons			20 neurons		
		Min	Max	Mean(SD)	Min	Max	Mean(SD)	Min	Max	Mean(SD)
traingd	20	61.0	80.0	71.0±6.0	58.0	80.0	69.0±4.9	58.0	75.0	66.0± 4.6
traingdm	20	60.0	78.0	69.0±5.2	61.0	75.0	68±5.0	55.0	72.0	65.0± 4.5
traingda	20	54.0	77.0	67.0±5.6	58.0	75.0	65.0± 4.5	46.0	78.0	64.0± 7.2
trainrp	20	58.0	80.0	67.0±5.0	52.0	78.0	68.0± 5.9	51.0	73.0	65.0± 5.9
traincgb	20	57.0	75.0	68.0±4.7	59.0	72.0	67.0± 3.0	51.0	77.0	66.0± 6.4
traincgp	20	55.0	83.0	67.0±7.1	58.0	74.0	65.0±4.8	46.0	77.0	65.0± 7.2
traincgf	20	46.0	73.0	64.0±6.1	46.0	74.0	66.0±6.4	58.0	74.0	65.0±5.1
trainscg	20	61.0	78.0	68.0± 4.5	59.0	75.0	65.0±4.3	54.0	75.0	64.0±5.3
trainlm	20	55.0	72.0	64.0± 5.4	54.0	74.0	65.0± 6.0	45.0	72.0	62.0±7.3
trainbfg	20	55.0	83.0	67.0± 7.0	57.0	74.0	66.0±4.7	45.0	72.0	63.0± 7.2
trainoss	20	64.0	83.0	69.0± 5.0	51.0	77.0	66.0±4.2	46.0	72.0	63.0±6.2
traingdx	20	64.0	78.0	69.0±4.0	58.0	74.0	68.0± 4.5	49.0	74.0	65.0±6.3
trainbr	20	60.0	77.0	67.0±4.6	59.0	75.0	68.0±4.0	52.0	77.0	68.0±6.2

The results showed superiority of the network with 5 neurons in hidden layer. The networks with 10 and 20 neurons were at the next orders, respectively. Accordingly, based on the maximum values, the algorithms named Polak-Ribiere conjugate gradient, BFGS quasi-newton and one step secant in 5 neurons (83%), basic gradient descent in 10 neurons (80%) and gradient descent with adaptive in 20 neurons (78%) structures trained the networks with most accuracy percentage in diagnosis. However, based on mean values, the algorithms such as Basic gradient descent (71%), Basic gradient descent (69%) and Bayesian regularization (68%) were chosen, respectively (Table 3). Furthermore, the area under the ROC curve was computed for the best trained network on each algorithm in the three defined structures. Table 4 summarizes the results. Although all the area under the curves were statistically significant (p-value<0.001), 5 neurons structure represented better results than two other structures. Based on this criterion, the 20 and 10 neurons structures are at the next ranks, respectively. As a result, the algorithms such as Scaled Conjugate Gradient and BFGS quasi-Newton (the area= 0.905) in 5 neurons, Gradient Descent with Momentum (the area=0.863) in 20 neurons and Bayesian regularization (the area=0.835) in 10 neurons structures had the highest diagnosis power on our clinical dataset.

**Table 4:** Comparison of the thirteen batch learning algorithms based on the area under the ROC curve

Learning algorithm	Area Under the ROC curve		

	5 neurons	10 neurons	20 neurons
Traingd	0.837	0.823	0.810
traingdm	0.832	0.768	0.863
traingda	0.848	0.819	0.814
trainrp	0.827	0.824	0.753
traincgb	0.745	0.797	0.837
traincgp	0.865	0.785	0.809
traincgf	0.814	0.788	0.811
trainscg	0.905	0.768	0.824
trainlm	0.768	0.734	0.800
trainbfg	0.905	0.814	0.779
trainoss	0.859	0.784	0.765
traingdx	0.875	0.745	0.825
trainbr	0.811	0.835	0.817

## 4. Discussion

Present study was conducted to help the physicians to diagnose the type of thyroid tumor in patients with a primary diagnosis of thyroid tumor surgery. The performance of thirteen different batch learning algorithms on prediction’s accuracy was compared for this purpose. This subject has not been sufficiently investigated in a single study. Therefore, this study may be important technically. Some recent researches investigated a subset of these algorithms (Koçer & Canal, 2011; Ramos-Pollán, Guevara-López, & Oliveira, 2012) or compared batch learning algorithms with online algorithms (Randall Wilsona & Martinez, 2003; Duchi & Singer, 2009; Perez-Suay, Francesc, Arevalillo-Herraez, & Jesus, 2013). Furthermore, the applications of these learning algorithms on other clinical problems such as image classification received more attention than cancers’ type diagnosis (Steven, Jinz, Zhuy & Lyuy, 2006).

In addition, since initial diagnosis of tumor type (malignancy/benign) affects type of surgery (subtotal or total lobectomy), results of this study may be noteworthy clinically. There are few studies which worked on modeling type of tumor or other diseases related to thyroid glands based on affecting factors. Some recent researches in this field used classic statistical methods such as logistic or linear regression analysis to model the relations among the factors (Lee & Kwak, 2010; Lima, Neto, Tambascia & Wittmann, 2013; Zou et al., 2013). For modeling with soft computing techniques, some studies applied neural network method to model the relations among the factors but not with the purpose of comparing different learning methods (Sarasvathi & Santhakumaran, 2011; Zhu et al., 2013; Bastias, Horvath, Baesler & Silva, 2011; Gharehchopogh, Molany & Mokri, 2013; Shukla, Tiwari, Kaur & Janghel, 2009; Ozyilmaz & Yildirim, 2002; Zhang & Berardi, 1998). However in our primary research in this field, three different methods of classification in data mining techniques had been compared on a subset of this dataset (Pourahmad, Azad, Paydar & Abbasi, 2012).

According to the text, result of FNA test as the preoperative diagnostic criterion may has some significant mistakes (Zhang & Berardi, 1998). In this study, result of FNA test compared with actual tumor type after surgery showed 63 percent accuracy in diagnosis. This is in agreement with other clinical texts. Whereas the represented modeling process in this study increased this accuracy rate up to at least 75 percent on favorites algorithms.

Furthermore, increasing neurons in hidden layer usually leads to better learning (Fausett, 1994) but our results did not confirm this fact.

At the end, although the algorithms offered acceptable and almost similar results in present study, work on larger dataset is recommended to achieve further opportunities of comparisons and derive more powerful diagnostic models in this medical problem.
